# Variation of Mass Effect After Using a Flow Diverter With Adjunctive Coil Embolization for Symptomatic Unruptured Large and Giant Intracranial Aneurysms

**DOI:** 10.3389/fneur.2019.01191

**Published:** 2019-11-12

**Authors:** Zhongxiao Wang, Zhongbin Tian, Wenqiang Li, Jiejun Wang, Wei Zhu, Mingqi Zhang, Ying Zhang, Jian Liu, Kun Wang, Yisen Zhang, Xinjian Yang

**Affiliations:** Department of Interventional Neuroradiology, Beijing Neurosurgical Institute and Beijing Tian Tan Hospital, Capital Medical University, Beijing, China

**Keywords:** aneurysm, mass effect, flow diverter (FD), coil embolization, endovsacular therapy

## Abstract

**Background:** Mass effect associated with large or giant aneurysms is an intractable problem for traditional endovascular treatments. Preventing recurrence of aneurysms requires dense coiling, which may aggravate the mass effect. However, the flow diverter (FD) is a new device that avoids the need for dense coiling. This study was performed to investigate whether use of FDs with adjunctive coil embolization can relieve the aneurysmal mass effect and to explore the factors that affect the variation of compressional symptoms.

**Methods:** We retrospectively evaluated patients with compressional symptoms caused by unruptured aneurysms who underwent endovascular treatment with an FD with adjunctive coil embolization at our center from January 2015 to December 2017. Imaging follow-up included digital subtraction angiography (DSA) ranging from 11 to 14 months and magnetic resonance imaging (MRI) ranging from 24 to 30 months; the former was used to evaluate the intracavitary volume, and the latter was used to measure the variation of the mass effect. Follow-up physical examinations were performed to observe variations of symptoms.

**Results:** In total, 22 patients with 22 aneurysms were treated by an FD combined with coil embolization. All 22 patients underwent the last clinical follow-up. Regarding compressional symptoms, 12 (54.54%) patients showed improvement, 6 (27.27%) were fully recovered, and 6 (27.27%) showed improvement but with incomplete cranial palsy. However, five (22.72%) patients showed no change, four (18.18%) showed worsening symptoms compared with their preoperative state, and one (4.55%) died of delayed rupture. Seventeen of the 22 patients underwent MRI. Of these 17 patients, the aneurysm shrank in 13 (76.47%) and no significant change occurred in 4 (23.53%). In the multivariate analysis, a short duration from symptom occurrence to treatment (*p* = 0.03) and younger patient age (*p* = 0.038) were statistically significant factors benefiting symptom improvement, and shrinkage of the aneurysm was associated with favorable clinical outcomes (*p* = 0.006).

**Conclusions:** Use of the FD with adjunctive loose coil embolization might help to alleviate the compressional symptoms caused by intracranial aneurysms. Shrinkage of the aneurysm, a short duration of symptoms, and younger patient age might contribute to favorable outcomes of mass effect-related symptoms.

## Introduction

Intracranial aneurysms are abnormal bulges of brain arteries that may rupture and bleed, leading to subarachnoid hemorrhage (1). Endovascular techniques have been proven effective and safe in the management of intracranial aneurysms and have thus become the preferred treatment strategy. However, traditional endovascular techniques are inferior for the treatment of large or giant aneurysms, which in some locations may induce compressional symptoms related to cranial nerves II, III, V, and VI, leading to oculomotor nerve palsy (ONP), visual loss, diplopia, and facial numbness (2–4). The classic intervention relies on coils for dense embolization in the aneurysmal sac, promoting thrombosis; however, if complete occlusion is achieved, the cranial palsy caused by the mass effect may become aggravated. The flow diverter (FD) provides a new solution to this intractable situation. FDs are woven stents constructed with high mesh density. They promote aneurysm occlusion by endoluminal reconstruction of the parent artery and redirection of blood flow away from the aneurysm (5, 6). The Pipeline embolization device (PED; ev3 Neurovascular, Irvine, CA, USA), which is one of the FDs that has been approved by the Food and Drug Administration, is the most commonly used FD at our center. Because of the excellent flow diverting force, the quantity of coils can be decreased during the intervention, making it possible to reduce the size of the aneurysm; the aneurysm may thus physically shrink by avoiding dense packing of the coil in the aneurysm (6–9). Numerous studies have revealed shrinkage of aneurysms and recovery of compressive cranial nerve symptoms following PED placement in cerebral aneurysms; however, almost all of these studies involved the use of a PED without coils (7–9). In other research, the complete absence of coils was found to be associated with a risk of delayed rupture; therefore, for giant aneurysms, it may be safer to use a PED with an appropriate quantity of loosely packed coils (10–12). One study showed that an FD with adjunctive coils may cause variations in the morphology of the aneurysm, both positively and negatively influencing the evolution of the symptoms (3, 7). However, the effect of a PED with loose packing on compressional symptoms caused by aneurysms is unclear. This study was performed to determine whether aneurysms can shrink after treatment with a PED with adjunctive coil embolization and identify the predictive factors for recovery of compressional symptoms.

## Materials and Methods

### Patients

We retrospectively collected the data of patients with symptomatic unruptured aneurysms who underwent endovascular treatment with a PED at our center from January 2015 to December 2017. Patients were included in this study if their symptoms were related to mass effect caused by a saccular aneurysm of the anterior circulation and they had objective neurological signs, the symptomatic aneurysm did not rupture before treatment, they received interventional treatment by a PED with adjunctive coil embolization, and their radiographic records included digital subtraction angiography (DSA).

We retrospectively reviewed the patients' hospital records to gather their clinical profiles, aneurysm characteristics, interventional details, and in-hospital complications. Demographic data including age, sex, smoking history, alcohol drinking history, hypertension, and diabetes mellitus were routinely collected. We paid close attention to the duration of symptoms (i.e., span of time from symptom occurrence to treatment). The patients were divided into two groups according to the variation of their symptoms at follow-up: improved and unimproved. Finally, both the identification of neurological signs and the collection of clinical information were performed by two investigators independently, and disagreements were resolved through discussion with another experienced neuroradiologist.

The Ethics Committee of Beijing Tian Tan Hospital approved this study. Written informed consent was obtained from each patient.

### Treatment Strategy

Our treatment strategy involved placement of a PED plus an appropriate quantity of loosely packed coils, which accelerates thrombogenesis in the sac, to reduce the risk of hemorrhage (12, 13). Moreover, we avoided dense packing to provide an opportunity for shrinkage of the aneurysm. The density of embolization was defined as the ratio of the volume of the coils to the volume of the aneurysm. Our previous report (14) describes the details of the packing strategy used in our center. In that study, we maintained an intracavity embolic density of <12% with reference to the earlier experiences of other investigators (15, 16), and we used this same criterion in the present study.

The volume of the coils was calculated as π × [(diameter of coil/2) × 2] × coil length (14). Each individual patient's aneurysm volume was scaled using Geomagic Studio version 12.0 (Geomagic Inc., Cary, NC, USA).

### Endovascular Procedure

All procedures were performed with the patient under general anesthesia and via a standard transfemoral approach with full heparinization. The specific dimensions of each PED were chosen after quantitation of the size of the aneurysmal neck and parent vessel diameter. Detachable coils were used during the procedure (Axium coils; Cordis, Fremont, CA, USA and MicroPlex coils; MicroVention-Terumo, Aliso Viejo, CA, USA).

Bilateral femoral artery puncture was performed to deliver a biaxial system. A 6-Fr long sheath, 5-Fr Navien (Medtronic, Dublin, Ireland), and Marksman microcatheter (ev3 Neurovascular, Irvine, CA, USA) were adopted to deliver the PED. On the other side, we navigated a second system consisting of a 5-Fr Envoy (Cordis) and a microcatheter (Echelon; Covidien, Dublin, Ireland) After positioning the Marksman microcatheter distal to the aneurysmal neck, we navigated the Echelon into the sac to subsequently deploy the coils for loose packing in the aneurysm.

### Antiplatelet Treatment and Anticoagulation

No pediatric patients were included in this study. All patients were premedicated with antiplatelet drugs for at least 5 days before the intervention. The patients received 75 mg of clopidogrel and 100 mg of aspirin daily. During the procedures, all patients were systemically heparinized with an activated clotting time of ≥200 s. Dual antiplatelet therapy was continued for at least 6 months after the procedure and aspirin was continued for life, in accordance with the standard embolism prophylaxis for intraluminal stent placement.

### Follow-Up

All patients were required to undergo at least two follow-up examinations, including DSA at 1 year postoperatively (first follow-up) and MRI at 2 years postoperatively (second-follow-up). Moreover, if the aneurysms were not occluded in the first DSA follow-up, the patient was recommended to undergo another DSA examination at 2 years.

The perioperative and first follow-up DSA findings were collected to affirm morphology and assess the transformation of the intracavitary condition. The preoperative and second follow-up MRI findings were used to determine whether any shrinkage of the aneurysms had occurred. Accordingly, we measured the largest diameter of the lesions on MRI and its perpendicular length, and quantified the involved lesions to calculate the volume. If the variation rate was >10%, variation of the aneurysm size was identified. The patients' clinical outcomes were measured by physical examination at the time of the outpatient MRI follow-up. The results were classified as either improved or unimproved, the latter of which included sustained and deteriorated symptoms compared with the pre-intervention status. Both the evaluation of clinical outcomes and the measurement of imaging parameters were performed by two investigators independently, and disagreements were resolved through discussion with another experienced neuroradiologist.

### Statistical Analysis

The statistical analysis was performed using SPSS 22.0 (IBM Corp., Armonk, NY, USA). A multivariate analysis, including the characteristics of both the patients and the aneurysms, was performed to determine the association of the clinical results with other factors. The characteristics of the patients and aneurysms in both groups were compared using the independent-samples *t*-test and Fisher's exact test, and *P* < 0.05 was considered to indicate statistical significance.

## Results

### Clinical Information and Aneurysm Characteristics

We identified 227 patients who underwent endovascular treatment with a PED with adjunctive coil embolization for intracranial aneurysms from September 2015 to December 2017, among whom 22 patients with 22 aneurysms causing compressional symptoms were included in the present study (Table 1). One patient (4.55%) had double aneurysms; the larger aneurysm (which probably caused the compression) was treated by a PED plus coil embolization. The smaller aneurysm was treated with an LVIS stent (MicroVention-Terumo) and was excluded from the analysis.

**Table 1:** Patient and aneurysm characteristics and follow-up outcomes.

**Case no**.	**Age**	**Duration**	**Size**	**Symptoms**	**DSA outcome**	**MRI outcome**	**Last clinical outcome**
1	28	≤1M	28.8	ONP	CO	Shrink	Improve
2	30	≤1M	27.2	VL	CO	Shrink	Improve
3	44	≤1M	36.9	VL	CO	Shrink	Improve
4	45	≤1M	12.1	VL	CO	Shrink	Improve
5	46	≤1M	13.3	ONP	CO	Shrink	Improve
6	50	≤1M	21.4	VL	CO	Shrink	Improve
7	51	≤1M	21.1	VL	CO	Shrink	Improve
8	56	≤1M	21.4	ONP+VL	CO	Shrink	Improve
9	59	≤1M	25.5	VL	CO	NA	Sustain
10	63	≤1M	26.1	ONP+VL	CO	Shrink	Aggravate
11	67	≤1M	29.2	VL	CO	Shrink	Aggravate
12	43	>1M	19.8	VL	CO	NA	Improve
13	44	>1M	29.9	VL	CO	Shrink	Improve
14	55	>1M	22.1	ONP	CO	Shrink	Improve
15	55	>1M	19.8	ONP+VL	CO	Shrink	Improve
16	57	>1M	21.4	VL	CO	Sustain	Sustain
17	59	>1M	32	VL	CO	Sustain	Aggravate
18	60	>1M	26.4	ONP+VL	CO	Sustain	Sustain
19	61	>1M	16.7	VL	CO	Sustain	Aggravate
20	62	>1M	36.7	VL	CO	NA	Sustain
21	69	>1M	21	VL	CO	NA	Sustain
22	72	>1M	21	VL	NA	NA	Aggravate

*DSA, digital subtraction angiography; MRI, magnetic resonance imaging; Duration, Time from symptoms onset to treatment; ONP, oculomotor nerve palsy; VL, visual loss; CO, complete occlusion; Sustain, no variation of symptoms; Aggravate, worsening of symptoms*.

The mean age of the 22 patients (17 women and 5 men) was 53.45 ± 11.44 years (range, 28–72 years). Nine (40.91%) aneurysms were located on the right side and 13 (59.09%) were located on the left side, and the mean maximum diameter was 24.5 ± 5.6 mm. The duration of symptoms was divided into two categories with 1 month as the boundary. Twelve (54.54%) patients received treatment within 1 month, and 10 (45.45%) patients received treatment beyond 1 month. There were 11 (50.00%) large aneurysms and 11 (50.00%) giant aneurysms. Fifteen (68.18%) patients had only visual loss, three (13.64%) had only ONP, and four (18.18%) had both ONP and visual loss before treatment (Table 2).

**Table 2:** Patient and aneurysm characteristics.

	**Value**	**Unit**
Age	53.45 ± 11.44	Year
Gender	5:17	
Size	24.5 ± 5.6	mm
Symptom		
ONP	3	
VL	15	
ONP+VL	4	
Total	22	
Mid follow-up	12.2 ± 0.7	Months
Last follow-up	25.5 ± 1.7	Months

*Data are presented as mean ± standard deviation or n. ONP, oculomotor nerve palsy; VL, visual loss*.

### Radiographic Follow-Up

**Figure 1:**
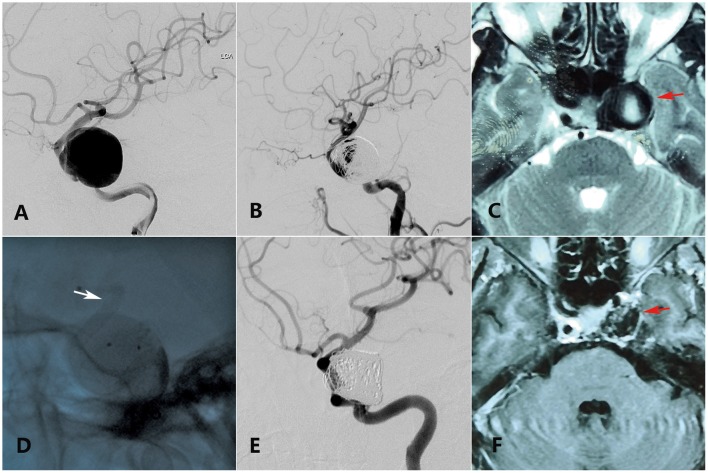
A 55-year-old woman with a giant ophthalmic segment ICA aneurysm presented with visual loss and incomplete ONP of the left side showing visual improvement merely in the last clinical follow-up. **(A)** Preoperative DSA shows a giant ophthalmic segment ICA aneurysm. **(B)** Immediate postoperative DSA shows loose embolization of aneurysm. **(C)** Preoperative axial-plane MRI shows an ophthalmic segment ICA aneurysm (red arrow). **(D)** Intraoperative DSA shows placement of a PED (white arrow) covering the aneurysmal neck. **(E)** Thirteen-month DSA shows variation of the coils in the sac. **(F)** Twenty-five-month axial-plane MRI shows shrinkage of the aneurysm (red arrow).

The angiographic follow-up consisted of the first DSA follow-up and a subsequent MRI follow-up. The former included all patients except one who died (total of 21/22, 95.45%) for a mean duration of 12.2 ± 0.7 months (range, 11–14 months). In this follow-up, all of the 21 aneurysms were completely occluded according to their DSA findings. The latter MRI follow-up included 17 of the 22 patients (65.38%) for a mean duration of 25.5 ± 1.7 months (range, 24–30 months). The images of these 17 aneurysms showed that no aneurysms had enlarged and that 13 (76.47%) had shrunk; however, 4 (23.53%) showed no significant change. Among these 13 patients whose aneurysm had shrunk compared with the preoperative size, 11 (84.62%) showed improvement at the last follow-up (Figure 1). However, none of the four patients without significant change on MRI improved compared with their preoperative state. Accordingly, aneurysm shrinkage (*p* = 0.006) may be important for relief of compressional symptoms.

### Clinical Follow-Up

**Figure 2:**
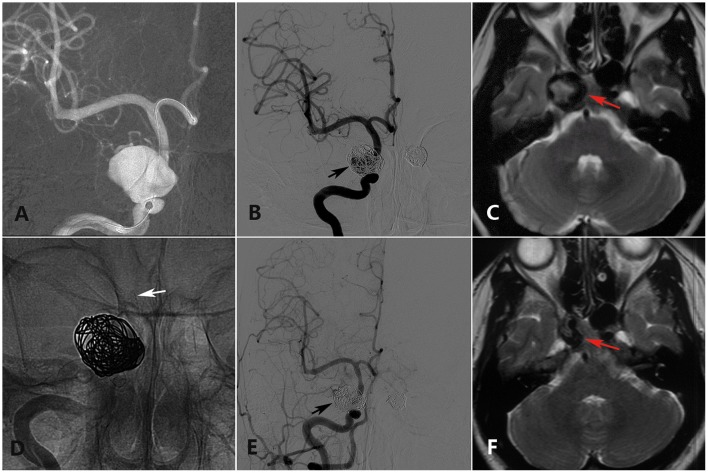
A 28-year-old woman with a double cavernous segment ICA aneurysm (the left-side aneurysm was excluded from analysis) presented with incomplete ONP of the right side showing full recovery in the last clinical follow-up. **(A)** Preoperative DSA shows a giant cavernous segment ICA aneurysm. **(B)** Immediate postoperative DSA shows loose embolization of the right aneurysm (black arrow). **(C)** Preoperative axial-plane MRI shows a cavernous segment ICA aneurysm (red arrow). **(D)** Intraoperative DSA shows placement of a PED (white arrow) covering the aneurysmal neck. **(E)** Twelve-month DSA shows collapse of the coils in the sac. **(F)** Twenty-four-month axial-plane MRI shows shrinkage of the aneurysm (red arrow).

At the last follow-up, 12 of the 22 patients (54.54%) showed improvement. Of these 12 patients, 6 (27.27%) were fully recovered (i.e., no abnormalities were present) (Figure 2) and 6 (27.27%) showed improvement but did not reach their premorbid level. On the one hand, among the 12 patients who improved, 9 (75.00%) had a treatment duration of ≤1 month. On the other hand, 8 of 10 patients (80.00%) who did not show improvement compared with their preoperative condition had a treatment duration exceeding 1 month. Accordingly, earlier treatment may have alleviated their compressional symptoms (*p* = 0.03). Five of the 22 patients (22.72%) showed no alterations and were thus categorized as sustaining. Four patients (18.18%) showed worsening compared with their preoperative status, including two (9.09%) with ischemic complications. All four of these patients presented with more severe compressional symptoms than their preoperative state. The remaining patient (4.55%) died of subarachnoid hemorrhage diagnosed at a local center 2 months postoperatively. According to the clinical follow-up, the mean age of the patients with improvement was 48.92 ± 11.43 years, and that of the patients with no improvement was 58.90 ± 9.23 years, indicating that a younger patient age may contribute to favorable clinical outcomes (*p* = 0.038).

The multivariate analysis showed that in regard to recovery of symptoms, aneurysm shrinkage was associated with favorable clinical outcomes (*p* = 0.006). The duration of symptoms (*p* = 0.03) and the patient's age at the time of the first treatment (*p* = 0.038) also had significant impacts (Table 3).

**Table 3:** Statistical results.

	**Improved**	**Unimproved**	***P*-value**
Age	48.9 ± 11.4	58.9 ± 9.2	0.038
Gender			0.323
Female	8	9	
Male	4	1	
MR			0.006
Shrink	11	2	
Sustain	0	4	
Duration			0.030
≤1 month	9	2	
>1 month	3	8	
Size			0.670
≤25 mm	7	4	
>25 mm	5	6	
Hypertension			1.000
Y	5	5	
N	7	5	
Diabetes			1.000
Y	2	1	
N	10	9	
Smoke			0.481
Y	2	0	
N	10	10	
Alcohol drink			1.000
Y	2	1	
N	10	9	

*Unimproved: includes sustained and aggravated. Improved: includes full recovery and improvement*.

## Discussion

Endovascular techniques have become the primary treatment strategy in the management of intracranial aneurysms (6, 17); however, such techniques are still inferior in the treatment of large or giant aneurysms with compressional symptoms. The traditional intervention relies on dense coil embolization within the sac, which promotes thrombosis (5). When complete occlusion is achieved, however, the cranial palsy caused by the mass effects may become aggravated. The PED provides a new solution to this intractable situation by decreasing the quantity of coils during the intervention, which allows for a reduction in the size of the aneurysm (7, 8). However, previous studies have shown that the complete absence of coils may increase the risk of delayed rupture (12, 13). Therefore, for large and giant aneurysms, it may be safer to use a PED with an appropriate quantity of loosely packed coils. However, the effect of the PED with loose packing on compressional symptoms following treatment of such aneurysms is unclear. In this respect, the following three findings of our study may be helpful: many of the aneurysms shrank after treatment with the PED and adjunctive loose coiling, the shrinkage of the aneurysms was associated with improvement of compressional symptoms, and the patient's age and duration of compression influenced the alleviation of the mass effect.

For large or giant aneurysms with compressional symptoms, a PED with adjunctive coil embolization has specific advantages. Earlier literature reported that the loss of pulsatility caused by aneurysm healing after traditional treatment had a certain probability of relieving symptoms (2, 5). However, the compression of the surrounding tissues by the aneurysm itself was considered to be unable to be resolved by the intervention, which may explain why compressional symptoms remain even after the aneurysm has been completely occluded. With the arising of FDs, coils no longer play an important role in interventional operations, making it possible to decrease the quantity of coils used to fill the aneurysm. Previous studies have revealed the feasibility and safety of using only a PED without coils. These studies indicate that the PED can effectively divert blood flow from large or giant aneurysms and can shrink the sac size during thrombosis, relieving the mass effect and even resolving the compressional symptoms (8, 9, 18). However, use of a PED alone may increase the risk of rupture after the intervention, especially for large or giant aneurysms, because the occlusion occurs over a delayed period (11, 12). Other investigators have stated that a coil-assisted PED can achieve early complete occlusion and endovascular reconstruction of the parent vessel, which may reduce the rate of re-treatment without inducing mass effect (11, 13). For these reasons, our center recommends the use of a PED with adjunctive coil embolization for large or giant aneurysms. Our results may indirectly support these conclusions because a lower rate of delayed rupture occurred in our center. Although delayed rupture of 1 aneurysm (4.55%) occurred in our study, 227 intracranial aneurysms were treated by a PED with adjunctive coils in our center from January 2015 to December 2017, and delayed rupture of only 2 (0.9%) occurred during this time. Moreover, 21 of 22 aneurysms (95.45%) in the present study achieved complete occlusion according to the DSA follow-up.

The development of thrombosis can gradually reduce the luminal volume of the sac after PED treatment, ending with complete occlusion. The subsequent thrombus organization has been considered the key to shrinkage of aneurysm sacs when coils are absent (7). In our study, patients who accepted treatment by a PED with adjunctive coil embolization exhibited shrinking of their aneurysm, which may be attributed to the lower density of embolization in the cavity. Previous studies did not focus on the embolization density in the aneurysm sac. Dense embolization is usually adopted to accelerate aneurysm occlusion and prevent recurrence. Slob et al. (19) reported that in dense embolization, the metal density in the sac was as high as 24% in some cases. Coils made by platinum usually have good ductility, but the high density of the coils forms a firm metal frame, propping up the aneurysm wall and making shrinkage difficult. Therefore, in the present study, loose embolization with a density of <12% reduced the metallic density of the aneurysm cavity, making it possible to shrink the aneurysm body.

Our data showed that the improvement of the clinical outcome was related to the shrinkage of the aneurysm (*p* = 0.006). Of concern, however, is that compressional symptoms remained in a few patients whose aneurysms obviously collapsed. A possible explanation for this phenomenon is that some of these patients did not receive timely treatment after symptom onset, and the relevant nerve may have thus been compressed too long to recover. The optimal treatment time window for such cranial nerve disorders has been mentioned in previous reports. Santillan et al. (20) described 11 patients with ONP due to a posterior communicating aneurysm, all of whom received interventional therapy within 1 week after symptom onset and 7 of whom experienced symptomatic relief during follow-up. The authors concluded that early management may be the strongest predictor of a positive clinical outcome (20). Brown et al. (21) had the same viewpoint. They published a study involving 45 patients treated from 2009 to 2013 and reported that 100% (11/11) of the patients experienced improvement if they were treated within 1 month of symptom onset and concluded that the time to treatment is an important consideration regardless of treatment modality. Similar results were obtained in our study: 11 patients received treatment within 1 month, and 9 of these 11 patients (81.82%) showed improvement at the last follow-up. Moreover, five of the six patients who achieved full recovery received treatment within 1 month. Eight of the 10 (80.00%) patients who showed no improvement received treatment beyond 1 month after their symptoms first occurred. Accordingly, the duration of compression may affect the recovery of nerve dysfunction.

There is a discrepancy between our results and published results. In our study, the age of the patients was considered to be a significant factor as suggested by the fact that the patient's age at the time of the first treatment had an impact on the clinical outcome (p = 0.038). For instance, the mean age of patients who showed improvement was 48.9 ± 11.4 years (range, 26–56 years), and that of patients who showed no improvement was 58.9 ± 9.2 years (range, 57–72 years). Additionally, among six patients aged >60 years, only one (16.67%) showed improvement. In contrast, among 16 patients aged ≤60 years, 11 (68.75%) showed improvement. To our knowledge, few previous studies of cranial nerve palsy have reported similar conclusions. However, in studies focusing on peripheral nerve damage, the patient's age is often found to have an impact on functional recovery (22). Accordingly, we conjecture that as age increases, the ability of cranial nerves (similar to peripheral nerves) to withstand damage gradually declines. More likely, older patients often do not notice diminution of vision as quickly as younger people. Thus, when they first visit a doctor, they tend to have more severe symptoms and a longer duration of symptoms than younger people, which is not conducive to improvement of the clinical outcome. For instance, of the six patients aged >60 years in our study, only two (33.33%) recognized their symptoms in a timely manner and received treatment within 1 month; the other four (66.67%) missed the optimal time window, and one patient was even treated beyond 1 year after the initial occurrence of symptoms. However, our statistical analysis showed no significant association between the patients' age and the duration of symptoms before treatment (*p* = 0.635). Moreover, one of the two older patients who received treatment in a timely manner showed full recovery. Therefore, although older patients are at a disadvantage in terms of recovery of compressional symptoms, we cannot conclude that they have no possibility of cure.

### Limitations

Large and giant aneurysms with obvious compression symptoms are rare, so the number of patients included in this study was relatively small. Additionally, some patients lacked MRI data, which may have led to bias of the statistical analysis. A prospective, multicenter investigation will be indispensable to explore the positive effects of treatment by PEDs with loose coiling on shrinkage of aneurysms and improvement of symptoms.

## Conclusion

Use of the FD with adjunctive loose coil embolization might help to alleviate the compressional symptoms caused by large or giant intracranial aneurysms. With the proper intracavity embolic density, the aneurysms would still have the potential to shrink. Furthermore, favorable outcomes of compressional symptoms might be associated with shrinkage of the aneurysm, the duration of symptoms, and the age of the patient.

## Data Availability Statement

The raw data supporting the conclusions of this manuscript will be made available by the authors, without undue reservation, to any qualified researcher.

## Ethics Statement

The studies involving human participants were reviewed and approved by The Ethics Committee of Beijing Tian Tan Hospital.

## Author Contributions

ZW and YisZ performed the manuscript writing. ZT, WL, JW, WZ, and MZ acquired the data. JL, KW, and YinZ analyzed and interpreted the data. XY conceived and designed the research, and handled funding and supervision.

### Conflict of Interest

The authors declare that the research was conducted in the absence of any commercial or financial relationships that could be construed as a potential conflict of interest.
